# Supplementary material for: Complexome profiling of the Chlamydomonas psb28 mutant reveals TEF5 as an early PSII assembly factor
Source: Plant Cell. 2025 Mar 17;37(6):koaf055. doi: 10.1093/plcell/koaf055 (PMC12164590; doi:10.1093/plcell/koaf055)
Supplement: koaf055_Supplementary_Data [file koaf055_supplementary_data.zip › psb28_interactive.zip!/plots/intensity/qualityPlots/peptides/OGD1_Cre12.g537200.t1.2.html]

### Cre12.g537200.t1.2

Trivial name: OGD1  
SeqCoverage [median]: 40.30  
Mass: 117.32 kDa  
  
 

| Sequence | MissedCleavage | Mass [Da] | wtIntensity | muIntensity | Score |
| --- | --- | --- | --- | --- | --- |
| AKQYYSEDHER | 1 | 1424.63 | 782.64 | 456.27 | 75.42 |
| AYQVLGHFAADLDPLR | 0 | 1784.92 | 1336.34 | 0.00 | 103.39 |
| DRGPRPPAEPEIK | 2 | 1460.77 | 797.91 | 5394.42 | 72.50 |
| DRGPRPPAEPEIKR | 3 | 1616.87 | 5911.68 | 25998.12 | 141.82 |
| DRIMQHLNAAFEGAK | 1 | 1699.85 | 0.00 | 1704.28 | 45.61 |
| EEVQQVR | 0 | 886.45 | 549.47 | 992.84 | 175.93 |
| EPIAHEGEYTGSGDVK | 0 | 1687.77 | 587.72 | 1463.33 | 60.97 |
| ERIETIDPVQFTK | 1 | 1574.83 | 479.95 | 2753.33 | 69.35 |
| FGLEGAESLIPGMK | 0 | 1447.74 | 5296.02 | 11131.52 | 173.64 |
| GPRPPAEPEIK | 1 | 1189.65 | 815.57 | 1200.90 | 81.52 |
| GRLNVLANVVR | 1 | 1209.73 | 0.00 | 7943.19 | 83.86 |
| HGHNEIDEPMFTQPLMYK | 0 | 2185.99 | 0.00 | 3732.59 | 50.83 |
| HSAQLYAER | 0 | 1073.53 | 1380.24 | 5194.43 | 91.87 |
| IETIDPVQFTK | 0 | 1289.69 | 25105.24 | 45564.89 | 153.97 |
| IMQHLNAAFEGAK | 0 | 1428.72 | 7385.30 | 18541.22 | 90.86 |
| IRNTGVPAELLR | 1 | 1337.78 | 4570.74 | 39103.48 | 68.68 |
| KPLIVMAPK | 1 | 995.62 | 0.00 | 2757.25 | 75.11 |
| KPMSQIFSEFAGK | 1 | 1468.74 | 347.81 | 0.00 | 59.90 |
| KPMSQIFSEFAGKEPIAHEGEYTGSGDVK | 2 | 3138.50 | 1398.37 | 12638.07 | 85.81 |
| LAMSPFTAAAISNQTVQESMR | 0 | 2252.09 | 6442.34 | 23969.86 | 131.37 |
| LIAEGTFTK | 0 | 978.54 | 1088.10 | 3616.05 | 119.41 |
| LIAEGTFTKEEVQQVR | 1 | 1846.98 | 12844.14 | 63183.39 | 159.31 |
| LIANALDVNYSGAI | 0 | 1432.76 | 2430.91 | 0.00 | 145.25 |
| LKDSFNEGTSITYLEELEER | 1 | 2372.14 | 1582.34 | 0.00 | 87.06 |
| LLLMIR | 0 | 757.49 | 0.00 | 4173.85 | 130.16 |
| LNVLANVVR | 0 | 996.61 | 16935.55 | 81240.16 | 180.70 |
| LSGQDVER | 0 | 902.45 | 29799.27 | 39331.80 | 188.98 |
| LSGQDVERGTFSHR | 1 | 1587.78 | 0.00 | 1039.47 | 52.58 |
| MPYAGRPPMAATATGFGEVHGK | 1 | 2245.08 | 890.41 | 11356.89 | 121.26 |
| NLDHGVTGEAMAESFDAFEK | 0 | 2166.95 | 3996.41 | 33974.55 | 132.76 |
| NLDHGVTGEAMAESFDAFEKGK | 1 | 2352.07 | 556.69 | 2960.81 | 120.41 |
| NTGVPAELLR | 0 | 1068.59 | 4451.15 | 16883.76 | 96.67 |
| QYYSEDHER | 0 | 1225.50 | 0.00 | 810.09 | 45.16 |
| RFGLEGAESLIPGMK | 1 | 1603.84 | 0.00 | 25208.10 | 94.72 |
| RVIMDDTGLTPK | 1 | 1344.71 | 269.33 | 0.00 | 82.36 |
| SPLYEFDDQPDDANIVGVR | 0 | 2149.00 | 364.70 | 1038.51 | 104.44 |
| STGLAITALPEDFAFHR | 0 | 1844.94 | 7404.66 | 44301.19 | 133.86 |
| TVIDTAADLGVQSVVIGMPHR | 0 | 2178.15 | 1967.31 | 24367.94 | 127.17 |
| VFYDLHDAR | 0 | 1134.55 | 1430.74 | 14165.64 | 71.35 |
| VFYDLHDAREK | 1 | 1391.68 | 0.00 | 1303.61 | 59.54 |
| VGEVAIVR | 0 | 841.50 | 40234.07 | 608267.00 | 182.26 |
| VIMDDTGLTPK | 0 | 1188.61 | 30321.12 | 52342.78 | 204.28 |
| YHKDPASVDR | 1 | 1186.57 | 17866.17 | 22915.55 | 174.40 |
| YHLGTSFNRPTVHGK | 1 | 1712.87 | 1729.17 | 2714.71 | 47.77 |
|  |  |  | 239349.6 | 1265735.9 |  |
